# Address before the Alumni Association of the Baltimore College of Dental Surgery

**Published:** 1878-05

**Authors:** S. J. Cockerille

**Affiliations:** President.


### ARTICLE III.

### *Address before Alumni Association of Baltimore College of Dental Surgery.*

BY S. J. COCKERILL, D. D. S., PRESIDENT.

GENTLEMEN OF THE ALUMNI ASSOCIATION:

In the vast domain of science and art, many subjects arise to attract us on such an occasion as this. The teachings we have received of our Alma Mater and the books we should take a pride in studying, have proved sources of gaining knowledge in *all* departments of learning, more accurate and lucid than could be contained in this form of address. Such an address to be acceptable, we are aware, should be presented in thought and language brilliant and glowing, and with all the beauties and intensity of eloquence. We have no such ambitious motive. Ours is *only* an earnest and determined effort to give fresh and vigorous impulse to the minds of our fellows in search after knowledge. In the effort to accomplish this, we must claim much from your indulgence.

The avocations of professional life afford but few leisure hours for literary pursuits. We indulge the hope that, not merit but the sincerity with which we offer this tribute of homage at the shrine of Alma Mater and our beloved profession, will commend approval.

A philosopher would tell us, that no art or science should be allowed pre-eminence, but the close observer of human nature must admit that the science or art, which is eagerly pursued for years, will appear of paramount importance. To prevent this is almost as difficult as passing through the eye of a needle. Some of our brethren have tried to convince us that this is an error. They tell us to rend asunder our Alma Mater and her sister institutions, and bury our glorious profession in oblivion. These brethren in their efforts to prove to the world they are truthful and honest, remind us of the atheist, who in his determination to be honest, and in his melancholy does not realize the fact he is praying to God. Thank God that these gentlemen though denying it themselves are praying for the success and prosperity of the profession. The hard study and actual physical labor necessary to perfect them in the profession dishearten and discourage them. With the average man it requires thirty years of hard work to become a perfect dentist—if it is ever done—but few will ever attain excellence.

We should guard against falling into the selfish, sordid, money-loving man, who follows the profession with ambition only for the half-dollar, and to keep a well stocked larder. The higher and nobler aims of the true artist and the man of science never enter the soul of such a creature. He forgets that the love of money is the root of all evil, and like

> "The sluggish owl that courts the night,
> Might check the eagle in his sun-ward flight,
> And think because to him it is not given
> No nobler bird can face the light of Heaven."

The money-lover is a curse to himself, a blot upon professional life, and may we not add, one of the most contemptible of God's creatures.

Anatomy, physiology, materia medica, pathology, metallurgy, botany and chemistry are accessory sciences to dentistry. The *Dentist* must acquire these sciences, and yet the acquisition of them all does not make the dentist. The dentist must educate the hand, ay, he must rise still higher, he must educate the heart.

The limited powers of the human mind have made it necessary to make a division of the sciences. Dentistry stands second to none other. What then is necessary to fully develop its importance, its dignity, its superiority to other branches of science? Our duty is not only to prepare ourselves for the profession, but to lend our best abilities and energies in inducing those who are yet to enter the ranks, to come thoroughly prepared.

How shall this be done? We who have studied anatomy, physiology and disease, know we constantly encounter names of Latin and Greek origin, therefore an understanding of these two languages should always precede the study of dentistry.

The study of dentistry is most favorable to the development of the mind and understanding, and to the refinement of the views of men. It does more to quicken the mind and elevate the soul than any other study.

How can we accomplish the greatest good to the profession? The answer is plain. Receive no student in our private offices who has not received a proper literary education, and discourage all from entering the profession who do not approach her portals thus armed. Just here allow us to say, we well know that nothing can supply the place of brains and character. We understand the difference between men ; one can accomplish more with an excavator and plugger, than another can do with best and most improved set of instruments. We believe in that education *only* which teaches how to think,. aye, we should have said how to work,—in that mental and physical training which produces the greatest possible developments.

Let students come to Alma Mater prepared for toil and research. Let students come impressed with the idea and belief that *they must* contribute to the advancement of the profession, and that a high and sure reward awaits them. Teach them to fulfill a bright destiny in some department of excellence, and the plaudits of a grateful profession will hail them as benefactors. Bring to our ranks genius combined with literary attainments. Teach them to devote themselves to literary pursuits as a professional duty.

The profession needs the soul-stirring, life-pervading influence of literary men. She cannot float safely by the side of her sister professions, and on the stream of progress upon which we would have her launch, without learning and science at the helm. The professional mind must be sharpened by collision. Let not our students be unprepared for this collision. The study and practice of dentistry incite the mind to intense activity. It is our duty to direct the younger minds to purposes of good, and not allow them to be perverted to mischief. Teach them to make their motto "upward and onward." The profession demands that we bring into her ranks educated genius, that truth may be discovered and fallacy detected. We must have varied learning, cultivated taste and high moral courage to attack and refute sophisms that may at any time endanger our onward and upward course.

Be governed by these principles and the future of dentistry will be better, brighter, nobler and higher as time goes on. If we will be determined, wise and true to ourselves, clinging to and laboring for the profession as she deserves, the day is not far distant when other professions and the public will look with admiring gaze to her beautiful, queenly and towering form, dispensing good to every body, preserving the health and beauty of all. To be wise is to be invulnerable.

Bring to the profession those, who by the light of their own genius will explore the heights beyond us. Truth must not be trodden under foot by careless observation and self-assertion. The annals of scientific research prove positively that the only way to keep comparatively free from errors, is to have accurate and pains taking observers and experimenters. Nothing short of educated genius will meet the needs of the profession and satisfy the seekers after truth.

Work is not noble from its results. It is work *itself* and the spirit in which it is done that constitute true nobility. True happiness is the wages of labor. Work and toil bring in view new truths which heretofore have been unsuspected. There is a dignity, a sense of relation between mental and physical labor in dentistry entirely wanting in other pursuits. Let students come to Alma Mater educated to work, so that they can concentrate the mind to the greatest advantage, and fix a separate and independent attention upon each particular branch of the profession. An educated trained mind sees things as they *are*, not as they are *supposed to be.* Every addition of a man of genius and learning to our ranks extends the limits of our dominions.

The pursuit of learning is noble and well worthy any amount of labor and toil. If prosecuted with ardor it rewards itself. It affords the highest pleasure, delight and gratification, that the mind can enjoy. Increase of appetite grows by what it feeds on. Her ways are ways of pleasantness and all her paths are peace.

It is the belief of the ignorant that a man to be eminent must confine his mind to one subject. The mind to be fully developed should be directed to every branch of art and science.

Our ranks must not be recruited from that class who obscure truth with careless observation; who are satisfied with unfaithful work; who in their laziness substitute inference for observation; who substitute falsehood for fact; who believe in words, not works; who will draw us into errors and confusion; who will exert a baneful influence upon our art and science: who pursue novelty and fancy.

Dentistry demands that her ranks be filled with educated genius, matchless skill, strong and determined will; an humble estimate of one's self; hatred of assumption and presumption; an anxious love of truth; with men who are determined to do their duty, and are not afraid of work and toil; with men who ever remember that he who acts as *duty* calls, shall *live*, though dead.

